# Author Correction: In situ formation of ZnO_*x*_ species for efficient propane dehydrogenation

**DOI:** 10.1038/s41586-021-04285-6

**Published:** 2022-01-06

**Authors:** Dan Zhao, Xinxin Tian, Dmitry E. Doronkin, Shanlei Han, Vita A. Kondratenko, Jan-Dierk Grunwaldt, Anna Perechodjuk, Thanh Huyen Vuong, Jabor Rabeah, Reinhard Eckelt, Uwe Rodemerck, David Linke, Guiyuan Jiang, Haijun Jiao, Evgenii V. Kondratenko

**Affiliations:** 1 State Key Laboratory of Heavy Oil Processing, China University of Petroleum, Beijing, P. R. China; 2 Leibniz-Institut für Katalyse e.V., Rostock, Germany; 3 Key Laboratory of Materials for Energy Conversion and Storage of Shanxi Province, Institute of Molecular Science, Shanxi University, Taiyuan, P. R. China; 4 Institute of Catalysis Research and Technology and Institute for Chemical Technology and Polymer Chemistry, Karlsruhe Institute of Technology (KIT), Karlsruhe, Germany

**Keywords:** Process chemistry, Materials for energy and catalysis, Heterogeneous catalysis

Correction to: *Nature* 10.1038/s41586-021-03923-3Published online 10 November 2021

In the version of this Article initially published, an error appeared in Extended Data Fig. 1a,b, where the content of the panels (plots and units, not axis labels) were inadvertently switched. The corrected version, with black data points in panel a, and blue, red and black data points in panel b, now appears correctly online.

The original Article has been corrected online.

